# Interventions, methods and outcome measures used in teaching evidence-based practice to healthcare students: an overview of systematic reviews

**DOI:** 10.1186/s12909-024-05259-8

**Published:** 2024-03-19

**Authors:** Lea D. Nielsen, Mette M. Løwe, Francisco Mansilla, Rene B. Jørgensen, Asviny Ramachandran, Bodil B. Noe, Heidi K. Egebæk

**Affiliations:** 1 Nursing Education & Department for Applied Health Science, University College South Denmark, Degnevej 17, 6705 Esbjerg Ø, Denmark; 2 Department of Oncology, Hospital of Lillebaelt, Beriderbakken 4, 7100 Vejle, Denmark; 3 Biomedical Laboratory Science & Department for Applied Health Science, University College South Denmark, Degnevej 17, 6705 Esbjerg Ø, Denmark; 4 Physiotherapy Education & Department for Applied Health Science, University College South Denmark, Degnevej 17, 6705 Esbjerg Ø, Denmark; 5 Occupational Therapy Education & Department for Applied Health Science, University College South Denmark, Degnevej 17, 6705 Esbjerg Ø, Denmark; 6 Department for Applied Health Science, University College South Denmark, Degnevej 17, 6705 Esbjerg Ø, Denmark; 7 Centre for Clinical Research and Prevention, Section for Health Promotion and Prevention, Bispebjerg and Frederiksberg Hospital, Nordre Fasanvej 57, 2000 Frederiksberg, Denmark

**Keywords:** MH "Students, Health occupations+", MH "Students, occupational therapy", MH "Students, physical therapy", MH "Students, Midwifery", “Students, Nursing"[Mesh], “Teaching"[Mesh], MH "Teaching methods+", "Evidence-based practice"[Mesh]

## Abstract

**Background:**

To fully implement the internationally acknowledged requirements for teaching in evidence-based practice, and support the student’s development of core competencies in evidence-based practice, educators at professional bachelor degree programs in healthcare need a systematic overview of evidence-based teaching and learning interventions. The purpose of this overview of systematic reviews was to summarize and synthesize the current evidence from systematic reviews on educational interventions being used by educators to teach evidence-based practice to professional bachelor-degree healthcare students and to identify the evidence-based practice-related learning outcomes used.

**Methods:**

An overview of systematic reviews. Four databases (PubMed/Medline, CINAHL, ERIC and the Cochrane library) were searched from May 2013 to January 25th, 2024. Additional sources were checked for unpublished or ongoing systematic reviews. Eligibility criteria included systematic reviews of studies among undergraduate nursing, physiotherapist, occupational therapist, midwife, nutrition and health, and biomedical laboratory science students, evaluating educational interventions aimed at teaching evidence-based practice in classroom or clinical practice setting, or a combination. Two authors independently performed initial eligibility screening of title/abstracts. Four authors independently performed full-text screening and assessed the quality of selected systematic reviews using standardized instruments. Data was extracted and synthesized using a narrative approach.

**Results:**

A total of 524 references were retrieved, and 6 systematic reviews (with a total of 39 primary studies) were included. Overlap between the systematic reviews was minimal. All the systematic reviews were of low methodological quality. Synthesis and analysis revealed a variety of teaching modalities and approaches. The outcomes were to some extent assessed in accordance with the Sicily group`s categories; “skills”, “attitude” and “knowledge”. Whereas “behaviors”, “reaction to educational experience”, “self-efficacy” and “benefits for the patient” were rarely used.

**Conclusions:**

Teaching evidence-based practice is widely used in undergraduate healthcare students and a variety of interventions are used and recognized. Not all categories of outcomes suggested by the Sicily group are used to evaluate outcomes of evidence-based practice teaching. There is a need for studies measuring the effect on outcomes in all the Sicily group categories, to enhance sustainability and transition of evidence-based practice competencies to the context of healthcare practice.

**Supplementary Information:**

The online version contains supplementary material available at 10.1186/s12909-024-05259-8.

## Background

Evidence-based practice (EBP) enhances the quality of healthcare, reduces the cost, improves patient outcomes, empowers clinicians, and is recognized as a problem-solving approach [1] that integrates the best available evidence with clinical expertise and patient preferences and values [2]. A recent scoping review of EBP and patient outcomes indicates that EBPs improve patient outcomes and yield a positive return of investment for hospitals and healthcare systems. The top outcomes measured were length of stay, mortality, patient compliance/adherence, readmissions, pneumonia and other infections, falls, morbidity, patient satisfaction, patient anxiety/ depression, patient complications and pain. The authors conclude that healthcare professionals have a professional and ethical responsibility to provide expert care which requires an evidence-based approach. Furthermore, educators must become competent in EBP methodology [3].

According to the Sicily statement group, teaching and practicing EBP requires a 5-step approach: 1) pose an answerable clinical question (Ask), 2) search and retrieve relevant evidence (Search), 3) critically appraise the evidence for validity and clinical importance (Appraise), 4) applicate the results in practice by integrating the evidence with clinical expertise, patient preferences and values to make a clinical decision (Integrate), and 5) evaluate the change or outcome (Evaluate /Assess) [4, 5]. Thus, according to the World Health Organization, educators, e.g., within undergraduate healthcare education, play a vital role by “integrating evidence-based teaching and learning processes, and helping learners interpret and apply evidence in their clinical learning experiences” [6].

A scoping review by Larsen et al. of 81 studies on interventions for teaching EBP within Professional bachelor-degree healthcare programs (PBHP) (in English undergraduate/ bachelor) shows that the majority of EBP teaching interventions include the first four steps, but the fifth step “evaluate/assess” is less often applied [5]. PBHP include bachelor-degree programs characterized by combined theoretical education and clinical training within nursing, physiotherapy, occupational therapy, radiography, and biomedical laboratory students., Furthermore, an overview of systematic reviews focusing on practicing healthcare professionals EBP competencies testifies that although graduates may have moderate to high level of self-reported EBP knowledge, skills, attitudes, and beliefs, this does not translate into their subsequent EBP implementation [7]. Although this cannot be seen as direct evidence of inadequate EBP teaching during undergraduate education, it is irrefutable that insufficient EBP competencies among clinicians across healthcare disciplines impedes their efforts to attain highest care quality and improved patient outcomes in clinical practice after graduation.

Research shows that teaching about EBP includes different types of modalities. An overview of systematic reviews, published by Young et al. in 2014 [8] and updated by Bala et al. in 2021 [9], synthesizes the effects of EBP teaching interventions including under- and post graduate health care professionals, the majority being medical students. They find that multifaceted interventions with a combination of lectures, computer lab sessions, small group discussion, journal clubs, use of current clinical issues, portfolios and assignments lead to improvement in students’ EBP knowledge, skills, attitudes, and behaviors compared to single interventions or no interventions [8, 9]. Larsen et al. find that within PBHP, collaboration with clinical practice is the second most frequently used intervention for teaching EBP and most often involves four or all five steps of the EBP teaching approach [5]. The use of clinically integrated teaching in EBP is only sparsely identified in the overviews by Young et al. and Bala et al. [8, 9]. Therefore, the evidence obtained within Bachelor of Medicine which is a theoretical education [10], may not be directly transferable for use in PBHP which combines theoretical and mandatory clinical education [11].

Since the overview by Young et al. [8], several reviews of interventions for teaching EBP used within PBHP have been published [5, 12–14].

We therefore wanted to explore the newest evidence for teaching EBP focusing on PBHP as these programs are characterized by a large proportion of clinical teaching. These healthcare professions are certified through a PBHP at a level corresponding to a University Bachelor Degree, but with strong focus on professional practice by combining theoretical studies with mandatory clinical teaching. In Denmark, almost half of PBHP take place in clinical practice. These applied science programs qualify “the students to independently analyze, evaluate and reflect on problems in order to carry out practice-based, complex, and development-oriented job functions" [11]. Thus, both the purpose of these PBHP and the amount of clinical practice included in the educations contrast with for example medicine.

Thus, this overview, identifies the newest evidence for teaching EBP specifically within PBHP and by including reviews using quantitative and/or qualitative methods.

We believe that such an overview is important knowledge for educators to be able to take the EBP teaching for healthcare professions to a higher level. Also reviewing and describing EBP-related learning outcomes, categorizing them according to the seven assessment categories developed by the Sicily group [2], will be useful knowledge to educators in healthcare professions. These seven assessment categories for EBP learning including: Reaction to the educational experience, attitudes, self-efficacy, knowledge, skills, behaviors and benefits to patients, can be linked to the five-step EBP approach. E.g., reactions to the educational experience: did the educators teaching style enhance learners’ enthusiasm for asking questions? (Ask), self-efficacy: how well do learners think they critically appraise evidence? (Appraise), skills: can learners come to a reasonable interpretation of how to apply the evidence? (Integrate) [2]. Thus, this set of categories can be seen as a basic set of EBP-related learning outcomes to classify the impact from EBP educational interventions.

### Purpose and review questions

A systematic overview of which evidence-based teaching interventions and which EBP-related learning outcomes that are used will give teachers access to important knowledge on what to implement and how to evaluate EBP teaching.

Thus, the purpose of this overview is to synthesize the latest evidence from systematic reviews about EBP teaching interventions in PBHP. This overview adds to the existing evidence by focusing on systematic reviews that a) include qualitative and/ or quantitative studies regardless of design, b) are conducted among PBHP within nursing, physiotherapy, occupational therapy, midwifery, nutrition and health and biomedical laboratory science, and c) incorporate the Sicily group's 5-step approach and seven assessment categories when analyzing the EBP teaching interventions and EBP-related learning outcomes.

The questions of this overview of systematic reviews are:

1. Which educational interventions are described and used by educators to teach EBP to Professional Bachelor-degree healthcare students?
2. What EBP-related learning outcomes have been used to evaluate teaching interventions?

## Methods

The study protocol was guided by the Cochrane Handbook on Overviews of Reviews [15] and the review process was reported in accordance with The Preferred Reporting Items for Systematic Reviews and Meta-analyses (PRISMA) statement [16] when this was consistent with the Cochrane Handbook.

### Inclusion criteria

Eligible reviews fulfilled the inclusion criteria for publication type, population, intervention, and context (see Table 1). Failing a single inclusion criterion implied exclusion.

**Table 1:** Inclusion and exclusion criteria of systematic reviews in the overview

Criteria	Inclusion	Exclusion
Publication type	Systematic reviews that have a specification of: • research question • clarity on the scope of the review • criteria for which studies are eligible for inclusion • a comprehensive literature search and has analyzed the included studies to draw conclusions based on all the identified research in an impartial and objective manner, i.e., performed data extraction and provides a synthesis of data and a quality appraisal of all the included studies A comprehensive literature search includes as a minimum a search in at least 2 databases relevant for the research question, provides keywords and /or search strategy, and justify publication restrictions (e.g., language)	Reviews and other study designs not fulfilling the definition of a systematic review
Population	Undergraduate / baccalaureate students from the disciplines of nursing, physiotherapist, occupational therapist, midwife, biomedical laboratory scientists and health & nutrition, including samples consisting of one healthcare discipline (e.g., nursing students) and samples consisting of several healthcare disciplines (e.g., nursing and physiotherapist students)	Undergraduate / baccalaureate students from other healthcare disciplines Post-graduate or continuous professional development students or health professionals (graduates) from present or other healthcare disciplines
Intervention	Reviews including studies on educational interventions with following characteristics: • interventions aimed at teaching one or more of the five steps of EBP in the Sicily statement; Ask, Search, Appraise, Integrate, Assess/evaluate (irrespective of format, mode or duration) and • that have been evaluated empirically with connection to EBP related outcomes	Reviews including studies that focused on issues other than methods for teaching EBP Or Reviews including studies on EBP educational interventions, that have not been evaluated empirically with connection to EBP related outcomes
Context	Reviews including studies conducted in classroom settings or clinical practice as part of the education, or in a combination of classroom and clinical practice settings	Other types of settings than classroom or clinical practice

### Search strategy

On January 25th 2024 a systematic search was conducted in; PubMed/Medline, CINAHL (EBSCOhost), ERIC (EBSCOhost) and the Cochrane library from May 2013 to January 25th, 2024 to identify systematic reviews published after the overview by Young et al. [8]. In collaboration with a research librarian, a search strategy of controlled vocabulary and free text terms related to systematic reviews, the student population, teaching interventions, teaching context, and evidence-based practice was developed (see Additional file 1). For each database, the search strategy was peer reviewed, revised, modified and subsequently pilot tested. No language restrictions were imposed.

To identify further eligible reviews, the following methods were used: Setting email alerts from the databases to provide weekly updates on new publications; backward and forward citation searching based on the included reviews by screening of reference lists and using the “cited by” and “similar results” function in PubMed and CINAHL; broad searching in Google Scholar (Advanced search), Prospero, JBI Evidence Synthesis and the OPEN Grey database; contacting experts in the field via email to first authors of included reviews, and by making queries via Twitter and Research Gate on any information on unpublished or ongoing reviews of relevance.

### Selection and quality appraisal process

Database search results were merged, duplicate records were removed, and title/abstract were initially screened via Covidence [17]. The assessment process was pilot tested by four authors independently assessing eligibility and methodological quality of one potential review followed by joint discussion to reach a common understanding of the criteria used. Two authors independently screened each title/abstract for compliance with the predefined eligibility criteria. Disagreements were resolved by a third author. Four authors were paired for full text screening, and each pair assessed independently 50% of the potentially relevant reviews for eligibility and methodological quality.

For quality appraisal, two independent authors used the AMSTAR-2 (A MeaSurement Tool to Assess systematic Reviews) for reviews including intervention studies [18] and the Joanna Briggs Institute Checklist for systematic reviews and research Synthesis (JBI checklist) [19] for reviews including both quantitative and qualitative or only qualitative studies. Uncertainties in assessments were resolved by requesting clarifying information from first authors of reviews and/or discussion with co-author to the present overview.

Overall methodological quality for included reviews was assessed using the overall confidence criteria of AMSTAR 2 based on scorings in seven critical domains [18] appraised as high (none or one non-critical flaw), moderate (more than one non-critical flaw), low (one critical weakness) or critically low (more than one critical weakness) [18]. For systematic reviews of qualitative studies [13, 20, 21] the critical domains of the AMSTAR 2, not specified in the JBI checklist, were added.

### Data extraction and synthesis process

Data were initially extracted by the first author, confirmed or rejected by the last author and finally discussed with the whole author group until consensus was reached.

Data extraction included 1) Information about the search and selection process according to the PRISMA statement [16, 22], 2) Characteristics of the systematic reviews inspired by a standard in the Cochrane Handbook (15), 3) A citation index inspired by Young et al. [8] used to illustrate overlap of primary studies in the included systematic reviews, and to ensure that data from each primary study were extracted only once [15], 4) Data on EBP teaching interventions and EBP-related outcomes. These data were extracted, reformatted (categorized inductively into two categories: “Collaboration interventions” and “ Educational interventions”) and presented as narrative summaries [15]. Data on outcome were categorized according to the seven assessment categories, defined by the Sicily group, to classify the impact from EBP educational interventions: Reaction to the educational experience, attitudes, self-efficacy, knowledge, skills, behaviors and benefits to patients [2]. When information under points 3 and 4 was missing, data from the abstracts of the primary study articles were reviewed.

## Results

### Results of the search

**Fig. 1:**
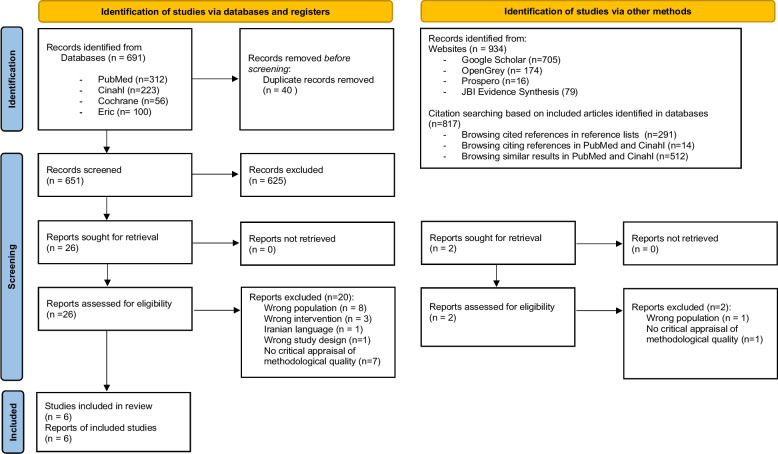
PRISMA flow diagram on search and selection of systematic reviews

The database search yielded 691 references after duplicates were removed. Title and abstract screening deemed 525 references irrelevant. Searching via other methods yielded two additional references. Out of 28 study reports assessed for eligibility 22 were excluded, leaving a total of six systematic reviews. Screening resulted in 100% agreement among the authors. Figure 1 details the search and selection process. Reviews that might seem relevant but did not meet the eligibility criteria [15], are listed in Additional file 2. One protocol for a potentially relevant review was identified as ongoing [23].

### Characteristics of included systematic reviews and overlap between them

The six systematic reviews originated from the Middle East, Asia, North America, Europe, Scandinavia, and Australia. Two out of six reviews did not identify themselves as systematic reviews but did fulfill this eligibility criteria [12, 20]. All six represented a total of 64 primary studies and a total population of 6649 students (see Table 2). However, five of the six systematic reviews contained a total of 17 primary studies not eligible to our overview focus (e.g., postgraduate students) (see Additional file 3). Results from these primary studies were not extracted. Of the remaining primary studies, six were included in two, and one was included in three systematic reviews. Data from these studies were extracted only once to avoid double-counting. Thus, the six systematic reviews represented a total of 39 primary studies and a total population of 3394 students. Nursing students represented 3280 of these. One sample of 58 nutrition and health students and one sample of 56 mixed nursing and midwife students were included but none from physiotherapy, occupational therapy, or biomedical laboratory scientists. The majority (*n* = 28) of the 39 primary studies had a quantitative design whereof 18 were quasi-experimental (see Additional file 4).

**Table 2:** Characteristics of included systematic reviews in the overview

**Authors, year/country** **Aim/Design**	**Search strategies (Databases searched, date-range of searches, last search up date)**	**N/n of student population** *N* = Total participants Included in review *n* = total participants included in present overview	**Review setting**	**Collaboration interventions**	**Educational Interventions (Teaching methods & Framing interventions)**	**N/n of primary studies** *N* = Total number of studies included in review *n* = total number of studies included in present overview **Design of studies**	**Quality appraisal**
Cui et al. 2018 [24] China Aim: compare the efficacy of EBN teaching versus traditional teaching Design: Systematic Review/meta synthesis of randomized controlled trials	Databases: Cochrane Central Register of Controlled Trials (CENTRAL) PubMed EMBASE Web of Science CINAHL Chinese BioMed Database (CBM) China National Knowledge Infrastructure (CNKI) WanFang Database Range: 2006–2016 Last search: 2017	1079/435	Classroom settings and clinical practice		Framing intervention: Nursing specialty Course: Introduce theoretical knowledge of EBP in Nursing (EBN); Make a skill training of EBN; Teach the lessons according to thinking of EBN Make EBN teaching plan; Master theory and skill of EBN, and Conduct EBP through case learning	9/4 Randomized Controlled Trial (RCT)	Cochrane Handbook for Systematic Reviews of Intervention version 5.1.0
Ghaffari et al. 2018 [25] Iran Aim: Systematically review the effectiveness of EBN education on the knowledge, skills, and attitudes of nursing student in Iran Design: Systematic review of quasi-experimental studies	Databases: SID Magiran Medlib Iranmedex PubMed Google Scholar CINAHL Range: 2011–2016 Last search: 2016	403/403	Classroom settings and clinical practice, but not specified in 6 out of 8 primary studies	- Clinical education with evidence-based method	Teaching methods: - Problem-based learning - Problem-solving approach - Research methodology teaching based on evidence-based care	8/8 Quasi-experimental studies	Risk of bias assessment was done manually by two researchers
Horntved et al. 2018 [20] Norway Aim: Identify the teaching strategies for EBP knowledge, and skills, currently used in undergraduate nursing education Design: A thematic literature review of qualitative studies	Databases: Medline Embase CINAHL ERIC, Academic Search Premier Range: 2006–2017 Last search: 2017	662 (incl. 68 lectures) /594	Classroom settings and clinical practice	Information literacy course Step-based Clinically integrated intervention: A four-stage intervention containing: information about voluntary participation in clinical research projects, education program related to EBP A Six-week program in reading and oral presentations of results from a research article related to a chosen field and topic of nursing practice Interdisciplinary collaboration: Collaboration between librarians, study skills advisors, and academics - Collaboration between faculty and library Clinical integrated interactive teaching interventions: - Integrated teaching activities to teach clinical strategies - Assignments - Assignments based on collaboration with health-care personnel in clinical practice - Assignment: choosing topics aimed at utilizing research and enhancing competence in the clinical practice context - Learning activities with oral presentations of the findings from the students’ studies in a clinical work setting - Participating in clinical projects and analyzing data with researchers - Clinical experts engaged in lectures - Conducting ‘mini’ research projects including an analysis of health needs of a particular patient group with a common problem or diagnosis - Participating and cooperating in clinical research projects	Framing interventions: - Establishing program related to the research process, incl. concept analysis, identifying evidence based quantitative and qualitative research, critical assessment, identifying discourses in documents, and practical implications of research as intervention programs - Experiential learning; often supplemented by collaborative group learning, such as partnerships for learning course content - Assignments, incl. carrying out a literature review, developing a proposal, facing a mock ethics committee, and collecting and analyzing data; supported by lectures, incl. via ‘Blackboard’ (virtual learning platform). Students presented their methodologic and analytic approaches on ‘Blackboard’ Teaching methods: - Lectures - Teaching sessions - Guided sessions - Essay writing - Assignments - Course assignment - Group work - Presentation Problem-based learning - Sharing information - Flipped classroom - Virtual simulation - Workshops - Group work - Seminars with discussions - Traditional teaching methods - Monthly sessions - Experiential teaching approaches - Assignment - Course assignments - Essay writing using different literature-based research methods	7/6 Qualitative and mixed-method studies	Critical Appraisal Skills Programme (CASP)
Patelarou et al. 2020 [12] Greece, Albania, Spain, Czech Republic, Poland, Slovenija, Italy Aim: Gather, assess, and synthesize evidence on educational interventions promoting evidence-based practice competencies in undergraduate nursing students Design: Scoping review/qualitative synthesis of quasi-experimental, mixed-method studies and randomized controlled trials	Databases: CINAHL PsychInfo Scopus PubMed EMBASE Cochrane Library Web of Science ProQuest EBSCOhost Springer Link and ScienceDirect + 5 non-English; CUIDEN, IBECS, SciELO, LILACS, and Polska Bibliografia Lekarska Range: 2004–2019 Last search: 2019	2280/1130	Classroom settings and clinical practice		Teaching methods: - Lectures - Small group works - Problem-based learning - Computer-based - learning - Team-based learning - Face-to-face intervention - Practice-based research questions - Voice-over PowerPoint, - Individual projects - Group projects - Seminar assignments and discussions - Lectures - Traditional instructor-led - Team-based learning - Virtual simulation - Pre-class assignments + Short individual pre-test followed by a group discussion and class on the pre-test, incl. instructor’s feedback - Face-to-face tutorials/online activities + a series of recommended readings and formative assessments to extend and consolidate student learning Framing interventions: Course on EBP Research and EBP course - Nursing research course Interactive: Evidence-based practice (EBP)-focused interactive teaching strategy five step EPB program of 4 weeks EBP program One year 2 phase educational program A 16-week research educational program 4 h cross-curricular EBP program 30 h EBP program,	20/13 Quasi-experimental, before and after studies	CASP
Ramis et al. 2019 [21] Australia Aim: Determine the effectiveness of EBP teaching strategies to undergraduate students with specific focus on efficacy of theory-based strategies Design: Systematic review of quasi-experimental and mixed methods studies	Databases: PubMed CINAHL Scopus ProQuest Health ERIC the Campbell Collaboration PsycINFO The New York Academy of Medicine ProQuest Dissertations Mednar Range: 2009–2016 Last search: 2016	719/346	Classroom settings		Teaching methods: - Assignment - Small groups - 2-h introductory lecture on principles, definition, steps of and resources needed for EBP - Dissemination - Q & A interactive discussions - Evidence-based internet teaching about EBP, incl. case-study based questions 30 min. video training on how to use evidence-based internet research tool - Interactive assignment - Q & A interactive discussions Framing Interventions; - Course based on Rogers’ diffusion of Innovation Model - Course based on interactive teaching intervention (1) problem identification and evidence synthesis 2) implementation strategy 3) dissemination - On Banduras self-efficacy, - On Rogers theory of diffusion - On Cognitive Apprenticeship theory (CAT) (scaffolding, exploring, - articulating, and reflecting) - On interactive teaching intervention (three steps: 1: problem identification and evidence synthesis; 2: implementation strategy; 3: dissemination)	5/3 Quasi-experimental	Risk of bias identified across studies
Wakibi et al. 2021 [13] Canada Aim: To synthesize, describe, and explore the evidence available to teach EBNP to undergraduate student nurses, so the students can continue to integrate EBNP in clinical settings upon becoming professional nurses Design: Systematic review with convergent qualitative synthesis of quantitative, qualitative and mixed methods studies	CINAHL MEDLINE EMBASE ERIC Web of Science Core Collection Range: 2008–2018 Last search: 2018	1506 /1380	Classroom and clinical settings	Clinically integrated interactive strategy: A strategy developed from model of diffusion of innovations and self-efficacy theory Interdisciplinary collaboration: -Collaboration between teaching institutions and clinical institutions -Collaboration between librarians, computer laboratory technicians and educators -Model CMBP for collaboration between university college and nursing practice Clinical integrated multi teaching intervention - Working on ward-based improvement areas following EBNP steps, facilitated throughout the process Working in small groups Presenting findings and recommendations to nurses and facilitators. Implementing changes in practice changes and evaluating changes - 1: Research course, 2: 6 days clinical practicum in clinical setting incorporating EBNP (happened twice; in the middle and end of semester): Two lectures on EBNP processes and concepts, working on hospital units in small groups, individual and group EBNP projects, small group conferences, presenting projects Clinical integrated teaching interventions: Clinical practicum in clinical setting incorporating EBNP Clinical integrated interactive teaching interventions: - EBNP project aiming at developing clinical practice guidelines - Describing EBNP project. EBNP project carried out in small groups throughout semester in partnership with clinical preceptors	Teaching methods: - Lectures - Seminars - Small group discussions - Quizzes - Assignments - Spiraling technique - Literature searching - Workshop - Small groups with facilitation from tutor and researcher - Seminar assignments and discussions - Group work - Individual work - Practical computer work - Journal clubs: students worked in small groups with nurses, specialists, charge nurses, and hospital directors - Presentations - Blended learning with lectures, small groups, online eRources - Blended learning: brainstorming, group work, and individual work - Summative and formative evaluation - Grade to evaluate students’ learning - Tests and examinations - Individual online learning - Learning divided into three component parts: 1. introducing research principles, 2. Teaching Evidence Based Nursing Practice (EBNP), 3: Understanding the application of EBNP concepts to practice - Pedagogical approach in teaching following a learning design (theoretical framework) with an underpinning scientific rationale in 3 stages: 1: Backward design, 2: Determining acceptable evidence, 3: Planning learning experience and instruction - Appraising articles in small groups, plenary discussions, lectures. Using a manual to guide article appraisal process. A final examination was taken - Self-directed learning of EBP basics (phase1) and workshops for critical appraisal of literature (phase2). At the end: presentations from groups Framing interventions: -Two-month EBNP learning strategy learning of EBP basics and critical appraisal of literature - Courses covering research methods and statistics, EBNP concepts and principles and it-skills - One-time EBNP course taught didactically covering research methods and statistics, EBNP concepts and principles - Research courses - EBP Course in EBP steps: 7-steps: fostering a spirit of inquiry and EBP culture, asking PICOT questions, searching for best evidence, critical appraisal, integrating evidence with clinical expertise and patient preferences to make clinical decisions, evaluating outcomes of EBP practice, and disseminating outcomes were described as useful steps - 15 weeks EBNP course, incl. biostatistics and epidemiology courses - 4 weeks EBNP course on reading, searching, and critically appraising evidence	15 /13 Qualitative, quantitative (before and after, quasi-experimental, cross-sectional studies), and mixed methods studies included	Joanna Briggs Institute + grading system Downe et al. 2009)

### Quality of systematic review

**Fig. 2:**
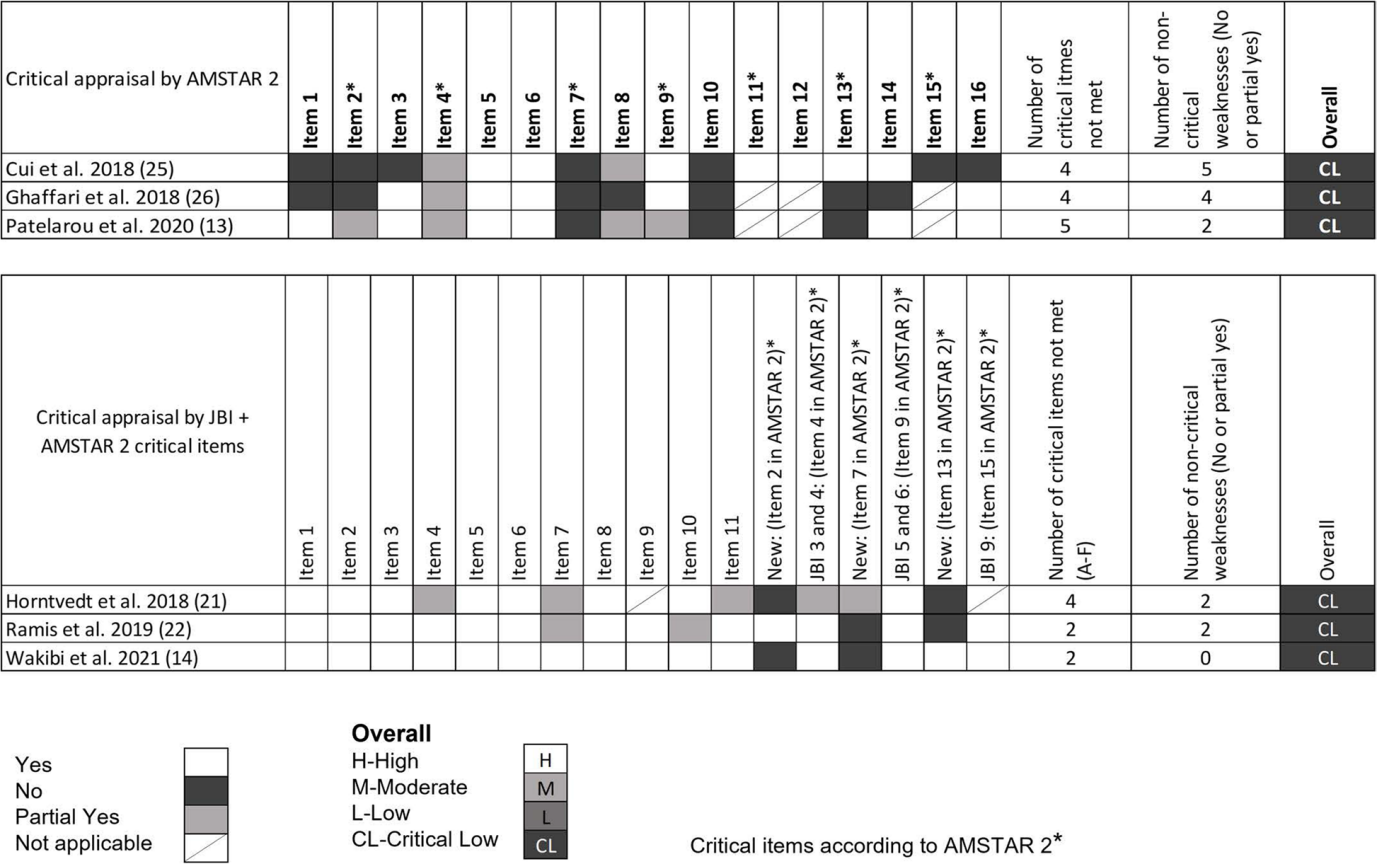
Overall methodological quality assessment for systematic reviews. Quantitative studies [12, 24, 25] were assessed following the AMSTAR 2 critical domain guidelines. Qualitative studies [13, 20, 21] were assessed following the JBI checklist. For overall classification, qualitative studies were also assessed with the following critical AMSTAR 2 domains not specified in the JBI checklist (item 2. is the protocol registered before commencement of the review, item 7. justification for excluding individual studies and item 13. consideration of risk of bias when interpreting the results of the review)

All the included systematic reviews were assessed as having critically low quality with 100% concordance between the two designed authors (see Fig. 2) [18]. The main reasons for the low quality of the reviews were a) not demonstrating a registered protocol prior to the review [13, 20, 24, 25], b) not providing a list of excluded studies with justification for exclusion [12, 13, 21, 24, 25] and c) not accounting for the quality of the individual studies when interpreting the result of the review [12, 20, 21, 25].

Missing reporting of sources of funding for primary studies and not describing the included studies in adequate detail were, most often, the two non-critical items of the AMSTAR 2 and the JBI checklist, not met.

Most of the included reviews did report research questions including components of PICO, performed study selection and data extraction in duplicate, used appropriate methods for combining studies and used satisfactory techniques for assessing risk of bias (see Fig. 2).

### Main findings from the systematic reviews

As illustrated in Table 2, this overview synthesizes evidence on a variety of approaches to promote EBP teaching in both classroom and clinical settings. The systematic reviews describe various interventions used for teaching in EBP, which can be summarized into two themes: Collaboration Interventions and Educational Interventions.

### Collaboration interventions to teach EBP

In general, the reviews point that interdisciplinary collaboration among health professionals and/or others e.g., librarian and professionals within information technologies is relevant when planning and teaching in EBP [13, 20].

Interdisciplinary collaboration was described as relevant when planning teaching in EBP [13, 20]. Specifically, regarding literature search Wakibi et al. found that collaboration between librarians, computer laboratory technicians and nurse educators enhanced students’ skills [13]. Also, in terms of creating transfer between EBP teaching and clinical practice, collaboration between faculty, library, clinical institutions, and teaching institutions was used [13, 20].

Regarding collaboration with clinical practice, Ghaffari et al. found that teaching EBP integrated in clinical education could promote students’ knowledge and skills [25]. Horntvedt et al. found that during a six-week course in clinical practice, students obtained better skills in reading research articles and orally presenting the findings to staff and fellow students [20]. Participation in clinical research projects combined with instructions in analyzing and discussing research findings also “led to a positive approach and EBP knowledge” [20]. Moreover, reading research articles during the clinical practice period enhances the students critical thinking skills. Furthermore, Horntvedt et al. mention, that students found it meaningful to conduct a “mini” – research project in clinical settings, as the identified evidence became relevant [20].

### Educational interventions

Educational interventions can be described as “Framing Interventions” understood as different ways to set up a framework for teaching EBP, and “ Teaching methods” understood as specific methods used when teaching EBP.

Various educational interventions were described in most reviews [12, 13, 20, 21]. According to Patelarou et al., no specific educational intervention regardless of framing and methods was in favor to “*increase knowledge, skills and competency as well as improve the beliefs, attitudes and behaviors of nursing students”* [12].

### Framing interventions

The approaches used to set up a framework for teaching EBP were labelled in different ways: programs, interactive teaching strategies, educational programs, courses etc. Approaches of various durations from hours to months were described as well as stepwise interventions [12, 13, 20, 21, 24, 25].

Some frameworks [13, 20, 21, 24] were based on the assessments categories described by the Sicily group [2] or based on theory [21] or as mentioned above clinically integrated [20]. Wakibi et al. identified interventions used to foster a spirit of inquiry and EBP culture reflecting the “5-step approach” of the Sicily group [4], asking PICOT questions, searching for best evidence, critical appraisal, integrating evidence with clinical expertise and patient preferences to make clinical decisions, evaluating outcomes of EBP practice, and disseminating outcomes useful [13]. Ramis et al. found that teaching interventions based on theory like Banduras self-efficacy or Roger’s theory of diffusion led to positive effects on students EBP knowledge and attitudes [21].

### Teaching methods

A variety of teaching methods were used such as, lectures [12, 13, 20], problem-based learning [12, 20, 25], group work, discussions [12, 13], and presentations [20] (see Table 2). The most effective method to achieve the skills required to practice EBP as described in the “5-step approach” by the Sicely group is a combination of different teaching methods like lectures, assignments, discussions, group works, and exams/tests.

Four systematic reviews identified such combinations or multifaceted approaches [12, 13, 20, 21]. Patelarou et al. states that “EBP education approaches should be blended” [12]. Thus, combining the use of video, voice-over, PowerPoint, problem-based learning, lectures, team-based learning, projects, and small groups were found in different studies. This combination had shown “to be effective” [12]. Similarly, Horntvedt et al. found that nursing students reported that various teaching methods improved their EBP knowledge and skills [20].

According to Ghaffari et al., including problem-based learning in teaching plans “improved the clinical care and performance of the students”, while the problem-solving approach “promoted student knowledge” [25]. Other teaching methods identified, e.g., flipped classroom [20] and virtual simulation [12, 20] were also characterized as useful interactive teaching interventions. Furthermore, face-to-face approaches seem “more effective” than online teaching interventions to enhance students’ research and appraisal skills and journal clubs enhance the students critically appraisal-skills [12].

As the reviews included in this overview primarily are based on qualitative, mixed methods as well as quasi-experimental studies and to a minor extent on randomized controlled trials (see Table 2) it is not possible to conclude of the most effective methods. However, a combination of methods and an innovative collaboration between librarians, information technology professionals and healthcare professionals seem the most effective approach to achieve EBP required skills.

### EBP-related outcomes

Most of the systematic reviews presented a wide array of outcome assessments applied in EBP research (See Table 3). Analyzing the outcomes according to the Sicily group’s assessment categories revealed that assessing “knowledge” (used in 19 out of 39 primary studies), “skills” (used in 18 out of 39 primary studies) and “attitude” (used in 17 out of 39) were by far the most frequently used assessment categories, whereas outcomes within the category of “behaviors” (used in eight studies) “reaction to educational experience” (in five studies), “self-efficacy” (in two studies), and “benefits for the patient” (in one study), were used to a far lesser extent. Additionally, outcomes, that we were not able to categorize within the seven assessment categories, were “future use” and “Global EBP competence”.

**Table 3:** EBP-related outcomes measured in the included systematic reviews according to the Sicily group's assessment categories

**Outcomes**	**Reaction to the educational experience**^**a**^	**Attitudes**	**Self-efficacy**	**Knowledge**	**Skills**	**Behaviours**	**Benefits to patients**	**Others**^B^
**Reviews**								
**Wakibi et al. 2021**^**c**^ [13]	15	1,4,6,7,8,9,12,13,15	15	1,2,6,7,8,13,14,15	4,9,13,14	8,9,12,15		Future use: 7,8
**Patelarou et al. 2020**^**c**^ [12]	40	29, 30, 32	43	29,30,32,43	26,29,30,32	32,43		Future use: 29 EBP competence: 29, 30
**Ramis et al. 2019** [21]		45		45				
**Horntvedt et al. 2018**^**c**^ [20]		28, 29,		28,34, 32	30,29,34,32			
**Ghaffari et al. 2018**^**c**^ [25]	5,7	3, 5		1,3,5	1,4	2,6	6	
**Cui et al. 2018** [24]					2,5,8,9			

The source numbers of the primary studies in each systematic review are based on the number of the source in the result tables of the review or reference list number (those using Vancouver)

Some of the primary studies were included in more than one systematic review (se Additional file 4), but are only presented from one review each in the table above

^a^Definitions on the Sicily assessment categories according to Tilson et al

• Reaction to educational experience: “refers to learners’ perspectives about the learning experience, including structural aspects (e.g., organization, presentation, content, teaching methods, materials, quality of instruction) and less tangible aspects such as support for learning” (2, p.3)

• Attitudes: “refers to the values ascribed by the learner to the importance and usefulness of EBP to inform clinical decision-making” (2, p.4)

• Self- Efficacy: “refers to people’s judgments regarding their ability to perform a certain activity (2, p.5)

• Knowledge: “refers to learners’ retention of facts and concepts about EBP” (2, p.5)

• Skills: “refer to the application of knowledge, ideally in a practical setting” (2, p.5)

• Behaviors as part of patient care: “refers to what learners actually do in practice. It is inclusive of all the processes that a clinician would use in the application of EBP, such as assessing patient circumstances, values, preferences, and goals along with identifying the clinician’s own competence relative to the patient’s needs in order to determine the focus of an answerable question (2, p.5)

• Benefits to patients: “refers to the impact of EBP educational interventions on the health of patients and communities” (2, p.6)

^b^The category “Others” represents outcomes that cannot be placed in any of the seven Sicily assessment categories

^c^Not all primary studies included in the review had result that could be (interpretated) placed in an assessment category

## Discussion

The purpose of this overview of systematic reviews was to collect and summarize evidence of the diversity of EBP teaching interventions and outcomes measured among professional bachelor- degree healthcare students.

Our results give an overview of “the state of the art” of using and measuring EBP in PBHP education. However, the quality of included systematic reviews was rated critically low. Thus, the result cannot support guidelines of best practice.

The analysis of the interventions and outcomes described in the 39 primary studies included in this overview, reveals a wide variety of teaching methods and interventions being used and described in the scientific literature on EBP teaching of PBHP students. The results show some evidence of the five step EBP approach in accordance with the inclusion criteria “interventions aimed at teaching one or more of the five EBP steps; Ask, Search, Appraise, Integrate, Assess/evaluate”. Most authors state, that the students´ EBP skills, attitudes and knowledge improved by almost any of the described methods and interventions. However, descriptions of how the improvements were measured were less frequent.

We evaluated the described outcome measures and assessments according to the seven categories proposed by the Sicily group and found that most assessments were on “attitudes”, “skills” and “knowledge”, sometimes on “behaviors” and very seldom on” reaction to educational experience”, “self-efficacy” and “benefits to the patients”. To our knowledge no systematic review or overview has made this evaluation on outcome categories before, but Bala et al. [9] also stated that knowledge, skills, and attitudes are the most common evaluated effects.

Comparing the outcomes measured between mainly medical [9] and nursing students, the most prevalent outcomes in both groups are knowledge, skills and attitudes around EBP. In contrast, measuring on the students´ patient care or on the impact of the EBP teaching on benefits for the patients is less prevalent. In contrast Wu et al.’s systematic review shows that among clinical nurses, educational interventions supporting implementation of EBP projects can change patient outcomes positively. However, they also conclude that direct causal evidence of the educational interventions is difficult to measure because of the diversity of EBP projects implemented [26]. Regarding EBP behavior the Sicily group recommend this category to be assessed by monitoring the frequency of the five step EBP approach, e.g., ASK questions about patients, APPRAISE evidence related to patient care, EVALUATE their EBP behavior and identified areas for improvement [2]. The results also showed evidence of student-clinician transition. “Future use” was identified in two systematic reviews [12, 13] and categorized as “others”. This outcome is not included in the seven Sicily categories. However, a systematic review of predictive modelling studies shows, that future use or the intention to use EBP after graduation are influenced by the students EBP familiarity, EBP capability beliefs, EBP attitudes and academic and clinical support [27].

Teaching and evaluating EBP needs to move beyond aiming at changes in knowledge, skills, and attitudes, but also start focusing on changing and assessing behavior, self-efficacy and benefit to the patients. We recommend doing this using validated tools for the assessment of outcomes and in prospective studies with longer follow-up periods, preferably evaluating the adoption of EBP in clinical settings bearing in mind, that best teaching practice happens across sectors and settings supported and supervised by multiple professions.

Based on a systematic review and international Delphi survey, a set of interprofessional EBP core competencies that details the competence content of each of the five steps has been published to inform curriculum development and benchmark EBP standards [28]. This consensus statement may be used by educators as a reference for both learning objectives and EBP content descriptions in future intervention research. The collaboration with clinical institutions and integration of EBP teaching components such as EBP assignments or participating in clinical research projects are important results. Specifically, in the light of the dialectic between theoretical and clinical education as a core characteristic of Professional bachelor-degree healthcare educations.

Our study has some limitations that need consideration when interpreting the results. A search in the EMBASE and Scopus databases was not added in the search strategy, although it might have been able to bring additional sources. Most of the 22 excluded reviews included primary studies among other levels/ healthcare groups of students or had not critically appraised their primary studies. This constitutes insufficient adherence to methodological guidelines for systematic reviews and limits the completeness of the reviews identified. Often, the result sections of the included reviews were poorly reported and made it necessary to extract some, but not always sufficient, information from the primary study abstracts. As the present study is an overview and not a new systematic review, we did not extract information from the result section in the primary studies. Thus, the comprehensiveness and applicability of the results of this overview are limited by the methodological limitations in the six included systematic reviews.

The existing evidence is based on different types of study designs. This heterogeneity is seen in all the included reviews. Thus, the present overview only conveys trends around the comparative effectiveness of the different ways to frame, or the methods used for teaching EBP. This can be seen as a weakness for the clarity and applicability of the overview results. Also, our protocol is unpublished, which may weaken the transparency of the overview approach, however our search strategies are available as additional material (see Additional file 1). In addition, the validity of data extraction can be discussed. We extracted data consecutively by the first and last author and if needed consensus was reached by discussion with the entire research group. This method might have been strengthened by using two blinded reviewers to extract data and present data with supporting kappa values.

The generalizability of the results of this overview is limited to undergraduate nursing students. Although, we consider it a strength that the results represent a broad international perspective on framing EBP teaching, as well as teaching methods and outcomes used among educators in EBP. Primary studies exist among occupational therapy and physiotherapy students [5, 29] but have not been systematically synthesized. However, the evidence is almost non-existent among midwife, nutrition and health and biomedical laboratory science students. This has implications for further research efforts because evidence from within these student populations is paramount for future proofing the quality assurance of clinical evidence-based healthcare practice.

Another implication is the need to compare how to frame the EBP teaching, and the methods used both inter-and mono professionally among these professional bachelor-degree students. Lastly, we support the recommendations of Bala et al. of using validated tools to increase the focus on measuring behavior change in clinical practice and patient outcomes, and to report in accordance with the GREET guidelines for educational intervention studies [9].

## Conclusion

This overview demonstrates a variety of approaches to promote EBP teaching among professional bachelor-degree healthcare students. Teaching EBP is based on collaboration with clinical practice and the use of different approaches to frame the teaching as well as different teaching methods. Furthermore, this overview has elucidated, that interventions often are evaluated according to changes in the student’s skills, knowledge and attitudes towards EBP, but very rarely on self-efficacy, behaviors, benefits to the patients or reaction to the educational experience as suggested by the Sicily group. This might indicate that educators need to move on to measure the effect of EBP on outcomes comprising all categories, which are important to enhance sustainable behavior and transition of knowledge into the context of practices where better healthcare education should have an impact. In our perspective these gaps in the EBP teaching are best met by focusing on more collaboration with clinical practice which is the context where the final endpoint of teaching EBP should be anchored and evaluated.

### Supplementary Information

**Supplementary Material 1.**

**Supplementary Material 2.**

**Supplementary Material 3.**

**Supplementary Material 4.**

## Data Availability

The datasets used an/or analyzed during the current study are available from the corresponding author on reasonable request.

## Abbreviations

EPB: Evidence-Based Practice
PBHP: Professional bachelor-degree healthcare programs
