# Supplementary material for: Interventions, methods and outcome measures used in teaching evidence-based practice to healthcare students: an overview of systematic reviews
Source: BMC Med Educ. 2024 Mar 19;24:306. doi: 10.1186/s12909-024-05259-8 (PMC10953117; doi:10.1186/s12909-024-05259-8)
Supplement: Supplementary file 1 — Supplementary Material 1. [file 12909_2024_5259_MOESM1_ESM.pdf]

Additional file 1. Search Strategy. Full documentation of search strategies in PubMed, Cinahl, PsycInfo, Eric and searching via other methods

**Databases**	**Set**	**Search terms**	**Last updated results**
PubMed/ Medline	1 Publication type	(((((((((("Systematic Review" [Publication Type]) OR "Meta-Analysis" [Publication Type]) OR "Review" [Publication Type]) OR (((("Systematic review") OR ("Meta analysis")) OR ("Meta-analysis")) OR ("Review")))))))	
	2 Population	(((((("Medical Laboratory Science/education"[Mesh]) OR ((("Clinical Dietetics") OR ("Clinical Dietician")) OR (((((((((("Students, Health Occupations"[Mesh]) OR "Physical Therapists/education"[Mesh]) OR "Occupational Therapists/education"[Mesh]) OR "Students, Nursing"[Mesh]) OR (((((((((((((((((((("Undergraduate health students") OR ("Allied health students")) OR ("Health occupations students")) OR ("health students")) OR ("Undergraduate nursing students")) OR ("Baccalaureate nursing students")) OR ("Undergraduate occupational students")) OR ("Baccalaureate occupational students")) OR ("Undergraduate physiotherapy students")) OR ("Baccalaureate physiotherapy students")) OR ("Undergraduate Medical Technician students")) OR ("Baccalaureate Medical Technician students")) OR ("Medical Technician students")) OR ("Undergraduate Midwife students")) OR ("Baccalaureate midwife students")) OR ("Midwife students")) OR ("Nutrition and health students")) OR ("Baccalaureate nutrition and health students")) OR ("Undergraduate nutrition and health students")) OR ("Dietitian")) OR ("Nutritionist")) OR ("Baccalaureate health students"))))) OR (((((((((((((((((((("Occupational therapist") OR ("Physiotherapist")) OR ("Nurse")) OR ("Physical therapist")) OR ("Biomedical laboratory scientist")) OR ("Medical Technologist")) OR ("Medical Laboratory Scientist")) OR ("Medical Technician")) OR ("Medical laboratory scientist")) OR ("Medical laboratory technologist")) OR ("Midwife")))	

		OR ("occupational therapists")) OR ("nurses")) OR ("physiotherapists")) OR ("midwifes")) OR ("dietitians")) OR ("nutritionists")) OR ("biomedical laboratory scientists")) OR ("medical technologists")) OR ("medical laboratory scientists")) OR ("medical technicians")) OR ("medical laboratory technologists")))))))))))))	
	3 Intervention	((("teaching models") OR ((((((((((((((((("Teaching"[Mesh]) OR "Learning"[Mesh])) OR "Curriculum"[Mesh]) OR (((((((((((((("teaching") OR ("teaching methods")) OR ("teaching strategies")) OR ("teaching interventions")) OR ("learning")) OR ("learning methods")) OR ("learning strategies")) OR ("learning interventions")) OR ("learning model")) OR ("educational strategies")) OR ("educational models")) OR ("educational interventions")) OR ("curriculum")) OR ("curricula designs"))))))))))	
	4 Context	((((((((((((((((((((((("Education, Nursing"[Mesh]) OR "Education, Nursing, Baccalaureate"[Mesh]) OR "Occupational Therapy/education"[Mesh]) OR "Physical Therapy Modalities/education"[Mesh]) OR "Midwifery/education"[Mesh]) OR "Medical Laboratory Personnel/education"[Mesh]) OR ((((((((((((("Nursing education") OR ("Physiotherapy education")) OR ("Occupational education")) OR ("Midwife education")) OR ("Biomedical education")) OR ("Nutrition and health education")) OR ("Evidence-based education")) OR ("Evidence-based nursing education")) OR ("Evidence-based occupational education")) OR ("Evidence-based midwife education")) OR ("Evidence-based nutrition and health education")) OR ("Evidence-based practice education")) OR ("Health education"))))) OR ("Evidence-based physiotherapy education")) OR ("nursing educations")) OR ("physiotherapy educations")) OR ("midwife educations")) OR ("occupational educations")) OR ("biomedical educations")) OR ("nutrition and health educations")) OR ("health educations")) OR ("evidence based occupational education")) OR ("evidence based physiotherapy education")) OR ("evidence based	

		midwife education")) OR ("evidence based nutrition and health education")))))))))	
	5<br>Outcomes	((((((((((("Evidence-Based Nursing"[Mesh]) OR "Evidence-Based Practice"[Mesh]) OR ((((((((((((("Evidence-based nursing practice") OR ("Evidence-based nursing")) OR ("Evidence-based physiotherapy")) OR ("Evidence-based occupational therapy")) OR ("Evidence-based physiotherapy practice")) OR ("Evidence-based occupational practice")) OR ("Evidence-based midwifery")) OR ("Evidence-based midwifery practice")) OR ("Evidence-based nutrition and health practice")) OR ("Evidence-based nutrition and health")) OR ("Evidence-based biomedical practice")) OR ("Evidence-based clinical practice")) OR ("Evidence-based practice")) OR ("Evidence-informed practice")) OR ("Evidence-based health care"))))) OR ("evidence based biomedical practice")) OR ("evidence based occupational practice")) OR (evidence based nutrition and health practice)) OR ("evidence-based nutrition")) OR ("evidence-based nutrition practice"))))))))	
	Filters	From May 1, 2013 to January 25, 2024	
		1 AND 2 AND 3 AND 4 AND 5	**312 (25.1.24)**

CINAHL (EBSOhost)	1<br>Publication type	"(MH "Systematic Review") OR (MH "Scoping Review") OR (MH "Meta Analysis") OR AB "Systematic review" OR "Meta analysis" OR "Meta-analysis" OR "Review" OR "scoping review"	
	2<br>population	"(MH "Students, Health Occupations+") OR (MH "Students, Occupational Therapy") OR (MH "Students, Physical Therapy") OR (MH "Students, Nursing, Baccalaureate+") OR (MH "Students, Nursing+") OR (MH "Students, Midwifery") OR (MH "Students, Medical Technology") OR AB "student occupational therapist" OR AB "student physical	

		therapist" OR AB "nursing student" OR AB midwifery student OR AB "medical technology student" OR AB ("nutrition and health student") OR AB "baccalaureate physiotherapy student" OR AB "baccalaureate occupational student" OR AB "baccalaureate nursing student" OR AB "baccalaureate midwife student" OR AB "Baccalaureate Medical Technician students" OR AB ("Baccalaureate nutrition and health students") OR AB "Undergraduate nursing students" OR AB "Undergraduate occupational students" OR AB "Undergraduate physiotherapy students" OR AB "Undergraduate Medical Technician students" OR AB ("Undergraduate nutrition and health students") OR AB "Undergraduate Midwife students" OR AB "Undergraduate health students" OR AB "Allied health students" OR AB "Health occupations students" OR AB "Baccalaureate health students" OR (MH "Physical Therapists/ED") OR (MH "Occupational Therapists/ED") OR (MH "Medical Technologists/ED") OR (MH "Midwives+/ED") OR (MH "Nurses+/ED") OR (MH "Dietitians/ED") OR AB "nurses" OR AB "occupational therapist" OR AB "physical therapist" OR AB "midwifes" OR AB ("nutrition and health") OR AB "nutritionist" OR AB "Biomedical laboratory scientist" OR AB "Medical Technologist" OR AB "Medical Laboratory Scientist" OR AB "Medical Technician" OR AB "Medical laboratory technologist" OR AB "Birth attendant" OR AB "Clinical Dietician" OR AB "Clinical Dietetics OR AB “occupational therapists” OR AB "physiotherapists" OR AB "nurse" OR AB "midwife" OR AB "birth attendants" OR AB "Medical Technologists" OR AB "Medical Laboratory Scientists" OR AB "Medical Technicians" OR AB "Medical laboratory technologists"	
	3 Intervention	"(MH "Teaching+") OR (MH "Teaching Methods+") OR (MH "Learning+") OR (MH "Learning Methods+") OR (MH "Curriculum+") OR (MH "Curriculum Development") OR (MH "Models, Educational/ED") OR AB "Teaching" OR "Teaching Methods" OR "Educational strategies" OR "Educational models" OR "Curricula designs" OR "Teaching strategies" OR "Learning strategies" OR "Teaching interventions"	

		OR "Learning interventions" OR "Learning" OR "educational interventions" OR "learning methods" OR "curriculum" OR "curriculum development"	
	4 Outcomes	"MH "Nursing Practice, Evidence-Based+") OR (MH "Physical Therapy Practice, Evidence-Based") OR (MH "Occupational Therapy Practice, Evidence-Based") OR (MH "Professional Practice, Evidence-Based+/ED") OR AB "Evidence-based nursing practice" OR “Evidence-based nursing ” OR "Evidence-based physiotherapy" OR "Evidence-based occupational therapy" OR "Evidence-based physiotherapy practice" OR "Evidence-based midwifery" OR “Evidence-based nutrition and health” OR "Evidence-based midwifery practice" OR "Evidence-based nutrition and health practice" OR "Evidence-based occupational practice" OR "Evidence-based biomedical practice" OR "Evidence-based clinical practice" OR “Evidence-based practice” OR “Evidence-informed practice” OR “Evidence-based health care” OR AB (evidence based nutrition and health practice) OR AB evidence based midwifery practice OR AB evidence based occupational practice OR AB evidence based physiotherapy practice OR AB (evidence based nutrition and health) OR AB evidence based midwifery	
	Filters	From May 1, 2013 to January 25, 2024	
		1 AND 2 AND 3 AND 4	**223 (25.1.24)**

ERIC (EBSCO host)	1 Publication type	DE "Meta Analysis" OR DE "Literature Reviews" OR "Systematic review" OR "Meta analysis" OR "Meta-analysis" OR "Review"	
	2 Population	"Undergraduate health students" OR "Allied health students" OR "Health occupations students" OR "health students" OR "Undergraduate nursing students" OR "Baccalaureate nursing students" OR "Undergraduate occupational students" OR "Baccalaureate occupational students" OR “occupational student” OR "Undergraduate physiotherapy students" OR "Baccalaureate physiotherapy students" OR “physiotherapy	

		students" OR "Undergraduate Medical Technician students" OR "Baccalaureate Medical Technician students" OR "Medical Technician students" OR "Undergraduate Midwife students" OR "Baccalaureate midwife students" OR "Midwife students" OR "Nutrition and health students" OR "Baccalaureate nutrition and health students" OR "Undergraduate nutrition and health students" OR "Dietitian" OR "Nutritionist" OR "Baccalaureate health students" OR "Occupational therapist" OR "Physiotherapist" OR "Nurse" OR "Physical therapist" OR "Biomedical laboratory scientist" OR "Medical Technologist" OR "Medical Laboratory Scientist" OR "Medical Technician" OR "Medical laboratory scientist" OR "Medical laboratory technologist" OR "Midwife" OR "occupational therapists" OR "nurses" OR "physiotherapists" OR "midwifes" OR "dietitians" OR "nutritionists" OR "biomedical laboratory scientists" OR "medical technologists" OR "medical laboratory scientists" OR "medical technicians" OR "medical laboratory technologists" OR DE "Nursing Students" OR DE "Allied Health Occupations Education"	
	3 Intervention	DE "Teaching Methods" OR DE "Learning Strategies" OR DE "Educational Strategies" OR DE "Learning" OR DE "Learning Activities" OR DE "Skill Development" AND DE "Educational Strategies" OR DE "Curriculum" OR DE "Curriculum Design" OR DE "Curriculum Development" OR "teaching" OR "teaching methods" OR "teaching strategies" OR "teaching interventions" OR "teaching models" OR "learning" OR "learning methods" OR "learning strategies" OR "learning interventions" OR "learning model" OR "educational strategies" OR "educational models" OR "educational interventions" OR "curriculum" OR "curricula designs"	
	4 Outcomes	"Evidence-based nursing practice" OR "Evidence-based nursing" OR "Evidence-based physiotherapy" OR "Evidence-based occupational therapy" OR "Evidence-based physiotherapy practice" OR "Evidence-based occupational practice" OR "Evidence-based midwifery" OR "Evidence-based midwifery practice" OR "Evidence-based nutrition	

		and health practice" OR "Evidence-based nutrition and health" OR "Evidence-based biomedical practice" OR "Evidence-based clinical practice" OR "Evidence-based practice" OR "Evidence-informed practice" OR "Evidence-based health care" OR "evidence based biomedical practice" OR "evidence based occupational practice" OR "evidence based nutrition and health practice" OR "evidence-based nutrition" OR "evidence-based nutrition practice" OR “evidence based nursing practice” OR “evidence based midwifery practice” OR “evidence based biomedical practice” OR DE "Evidence Based Practice" OR DE "Evidence Based Practice" OR “Nursing education" OR "Physiotherapy education" OR "Occupational education" OR "Midwife education" OR "Biomedical education" OR "Nutrition and health education" OR "Evidence-based education" OR "Evidence-based nursing education" OR "Evidence-based occupational education" OR "Evidence-based midwife education" OR "Evidence-based nutrition and health education" OR "Evidence-based practice education" OR "Health education" OR "Evidence-based physiotherapy education" OR “Evidence-based biomedical education” OR "nursing educations" OR "physiotherapy educations" OR "midwife educations" OR "occupational educations" OR "biomedical educations" OR "nutrition and health educations" OR "health educations" OR "evidence based occupational education" OR "evidence based physiotherapy education" OR "evidence based midwife education" OR "evidence based nutrition and health education" OR “evidence based biomedical education” OR DE "Nursing Education" OR DE "Physical Therapy" OR DE "Nursing Education" OR DE "Occupational Therapy" OR DE "Obstetrics" OR midwifery	
	Filters	From May 1, 2013 to May 10, 2022	
		1 AND 2 AND 3 AND 4	**100 (25.1.24)**

Cochrane	1	MeSH descriptor: [Teaching] explode all trees	
	2	MeSH descriptor: [Learning] explode all trees	
	3	MeSH descriptor: [Curriculum] explode all trees	

	4	("teaching" OR "teaching methods" OR "teaching strategies" OR "teaching interventions" OR "learning" OR "learning methods" OR "learning strategies" OR "learning interventions" OR "learning model" OR "educational strategies" OR "educational models" OR "educational interventions" OR "curriculum" OR "curricula designs"):ti,ab,kw (Word variations have been searched)	
	5	#1 OR #2 OR #3 OR #4	
	6	MeSH descriptor: [Education, Nursing] explode all trees	
	7	MeSH descriptor: [Education, Nursing, Baccalaureate] explode all trees	
	8	MeSH descriptor: [Occupational Therapy] explode all trees	
	9	MeSH descriptor: [Physical Therapy Modalities] explode all trees	
	10	MeSH descriptor: [Midwifery] explode all trees	
	11	MeSH descriptor: [Medical Laboratory Personnel] explode all trees	
	12	"Nursing education" OR "Physiotherapy education" OR "Occupational education" OR "Midwife education" OR "Biomedical education" OR "Nutrition and health education" OR "Evidence-based education" OR "Evidence-based nursing education" OR "Evidence-based occupational education" OR "Evidence-based midwife education" OR "Evidence-based nutrition and health education" OR "Evidence-based practice education" OR "Health education" OR "Evidence-based physiotherapy education" OR "nursing educations" OR "physiotherapy educations" OR "midwife educations" OR "occupational educations" OR "biomedical educations" OR "nutrition and health educations" OR "health educations" OR "evidence based occupational education" OR "evidence based physiotherapy education" OR "evidence based midwife education" OR "evidence based nutrition and health education"	
	13	#6 OR #7 OR #8 OR #9 OR #10 OR #11 OR #12	
	14	MeSH descriptor: [Evidence-Based Nursing] explode all trees	
	15	MeSH descriptor: [Evidence-Based Practice] explode all trees	
	16	("Evidence-based nursing practice" OR "Evidence-based nursing" OR "Evidence-based physiotherapy" OR "Evidence-based occupational therapy" OR "Evidence-based physiotherapy practice" OR "Evidence-based occupational practice" OR "Evidence-based midwifery" OR "Evidence-based midwifery practice" OR "Evidence-based	

		nutrition and health practice" OR "Evidence-based nutrition and health" OR "Evidence-based biomedical practice" OR "Evidence-based clinical practice" OR "Evidence-based practice" OR "Evidence-informed practice" OR "Evidence-based health care" OR "evidence based biomedical practice" OR "evidence based occupational practice" OR "evidence based nutrition and health practice" OR "evidence-based nutrition" OR "evidence-based nutrition practice"):ti,ab,kw (Word variations have been searched)	
	17	#14 OR #15 OR #16	
	18	MeSH descriptor: [Students, Health Occupations] explode all trees	
	19	MeSH descriptor: [Physical Therapists] explode all trees	
	20	MeSH descriptor: [Occupational Therapists] explode all trees	
	21	MeSH descriptor: [Students, Nursing] explode all trees	
	22	"Undergraduate health students" OR "Allied health students" OR "Health occupations students" OR "health students" OR "Undergraduate nursing students" OR "Baccalaureate nursing students" OR "Undergraduate occupational students" OR "Baccalaureate occupational students" OR "Undergraduate physiotherapy students" OR "Baccalaureate physiotherapy students" OR "Undergraduate Medical Technician students" OR "Baccalaureate Medical Technician students" OR "Medical Technician students" OR "Undergraduate Midwife students" OR "Baccalaureate midwife students" OR "Midwife students" OR "Nutrition and health students" OR "Baccalaureate nutrition and health students" OR "Undergraduate nutrition and health students" OR "Dietitian" OR "Nutritionist" OR "Baccalaureate health students" OR "Occupational therapist" OR "Physiotherapist" OR "Nurse" OR "Physical therapist" OR "Biomedical laboratory scientist" OR "Medical Technologist" OR "Medical Laboratory Scientist" OR "Medical Technician" OR "Medical laboratory scientist" OR "Medical laboratory technologist" OR "Midwife" OR "occupational therapists" OR "nurses" OR "physiotherapists" OR "midwifes" OR "dietitians" OR "nutritionists" OR "biomedical laboratory scientists" OR "medical technologists" OR "medical laboratory scientists" OR "medical technicians" OR "medical laboratory technologists"	
	23	#18 OR #19 OR #20 OR #21 OR #22	

	24	MeSH descriptor: [Systematic Review] explode all trees	
	25	MeSH descriptor: [Meta-Analysis] explode all trees	
	26	MeSH descriptor: [Review] explode all trees	
	27	"Systematic review" OR "Meta analysis" OR "Meta-analysis" OR "Review"	
	28	#24 OR #25 #26 OR #27	
	29	#5 AND #13 AND #17 AND #23 AND #28	
	30	MeSH descriptor: [Teaching] explode all trees	
	31	MeSH descriptor: [Learning] explode all trees	
	32	MeSH descriptor: [Curriculum] explode all trees	
	33	("teaching" OR "teaching methods" OR "teaching strategies" OR "teaching interventions" OR "learning" OR "learning methods" OR "learning strategies" OR "learning interventions" OR "learning model" OR "educational strategies" OR "educational models" OR "educational interventions" OR "curriculum" OR "curricula designs"):ti,ab,kw (Word variations have been searched)	
	34	#30 OR #31 OR #32 OR #33	
	35	MeSH descriptor: [Education, Nursing] explode all trees	
	36	MeSH descriptor: [Education, Nursing, Baccalaureate] explode all trees	
	37	MeSH descriptor: [Occupational Therapy] explode all trees	
	38	MeSH descriptor: [Physical Therapy Modalities] explode all trees	
	39	MeSH descriptor: [Midwifery] explode all trees	
	40	MeSH descriptor: [Medical Laboratory Personnel] explode all trees	
	41	"Nursing education" OR "Physiotherapy education" OR "Occupational education" OR "Midwife education" OR "Biomedical education" OR "Nutrition and health education" OR "Evidence-based education" OR "Evidence-based nursing education" OR "Evidence-based occupational education" OR "Evidence-based midwife education" OR "Evidence-based nutrition and health education" OR "Evidence-based practice education" OR "Health education" OR "Evidence-based physiotherapy education" OR "nursing educations" OR "physiotherapy educations" OR "midwife educations" OR "occupational educations" OR "biomedical educations" OR "nutrition and health educations" OR "health educations" OR "evidence based occupational education" OR "evidence based physiotherapy education" OR "evidence based midwife education" OR "evidence based nutrition and health education"	
	42	#35 OR #36 OR #37 OR #38 OR #39 OR #40 OR #41	

	43	MeSH descriptor: [Evidence-Based Nursing] explode all trees	
	44	MeSH descriptor: [Evidence-Based Practice] explode all trees	
	45	("Evidence-based nursing practice" OR "Evidence-based nursing" OR "Evidence-based physiotherapy" OR "Evidence-based occupational therapy" OR "Evidence-based physiotherapy practice" OR "Evidence-based occupational practice" OR "Evidence-based midwifery" OR "Evidence-based midwifery practice" OR "Evidence-based nutrition and health practice" OR "Evidence-based nutrition and health" OR "Evidence-based biomedical practice" OR "Evidence-based clinical practice" OR "Evidence-based practice" OR "Evidence-informed practice" OR "Evidence-based health care" OR "evidence based biomedical practice" OR "evidence based occupational practice" OR "evidence based nutrition and health practice" OR "evidence-based nutrition" OR "evidence-based nutrition practice"):ti,ab,kw (Word variations have been searched)	
	46	#43 OR #44 OR #45	
	47	MeSH descriptor: [Students, Health Occupations] explode all trees	
	48	MeSH descriptor: [Physical Therapists] explode all trees	
	49	MeSH descriptor: [Occupational Therapists] explode all trees	
	50	MeSH descriptor: [Students, Nursing] explode all trees	
	51	"Undergraduate health students" OR "Allied health students" OR "Health occupations students" OR "health students" OR "Undergraduate nursing students" OR "Baccalaureate nursing students" OR "Undergraduate occupational students" OR "Baccalaureate occupational students" OR "Undergraduate physiotherapy students" OR "Baccalaureate physiotherapy students" OR "Undergraduate Medical Technician students" OR "Baccalaureate Medical Technician students" OR "Medical Technician students" OR "Undergraduate Midwife students" OR "Baccalaureate midwife students" OR "Midwife students" OR "Nutrition and health students" OR "Baccalaureate nutrition and health students" OR "Undergraduate nutrition and health students" OR "Dietitian" OR "Nutritionist" OR "Baccalaureate health students" OR "Occupational therapist" OR "Physiotherapist" OR "Nurse" OR "Physical therapist" OR "Biomedical laboratory scientist" OR "Medical	

		Technologist" OR "Medical Laboratory Scientist" OR "Medical Technician" OR "Medical laboratory scientist" OR "Medical laboratory technologist" OR "Midwife" OR "occupational therapists" OR "nurses" OR "physiotherapists" OR "midwifes" OR "dietitians" OR "nutritionists" OR "biomedical laboratory scientists" OR "medical technologists" OR "medical laboratory scientists" OR "medical technicians" OR "medical laboratory technologists"	
	52	#47 OR #48 OR #49 OR #50 OR #51	
	53	MeSH descriptor: [Systematic Review] explode all trees	
	54	MeSH descriptor: [Meta-Analysis] explode all trees	
	55	MeSH descriptor: [Review] explode all trees	
	56	"Systematic review" OR "Meta analysis" OR "Meta-analysis" OR "Review"	
	57	#53 OR #54 #55 OR #56	
	58	#34 AND #42 AND #46 AND #52 AND #57	
	Filters	From May 1, 2013 to January 25, 2024	**56 (25.1.24)**

**Searching via other methods**		
Google Scholars (Advanced Search)	Evidence-based AND student AND review/ Evidence-Based AND reviews	**705 (10.5.22) Not updated January 2024 because the value is too small**
Open Grey	from 2013 to May 2021 using our PubMed search string and single or combined searches with the terms; evidence-based nursing, evidence-based physiotherapy, evidence-based occupational therapy, evidence-based education/ practice	**174 (18.5.21) Database closed in summer 2021**
Prospero	Mesh DESCRIPTOR Evidence-based Practice EXPLODE ALL TREES WHERE CD FROM 01/05/2013 TO 01/05/2022 AND Mesh DESCRIPTOR Education EXPLODE ALL TREES WHERE CD FROM 01/05/2013 TO 25/01/2024	**16 (25.1.24)**
JBI Evidence Synthesis	AB “Evidence-based practice” AND AB Education	**79 (25.1.24)**
