# Supplementary material for: Interventions, methods and outcome measures used in teaching evidence-based practice to healthcare students: an overview of systematic reviews
Source: BMC Med Educ. 2024 Mar 19;24:306. doi: 10.1186/s12909-024-05259-8 (PMC10953117; doi:10.1186/s12909-024-05259-8)
Supplement: Supplementary file 2 — Supplementary Material 2. [file 12909_2024_5259_MOESM2_ESM.pdf]

Additional file 2. Reviews that might appear to meet the inclusion criteria, but which were excluded including reason for exclusion

Study ID	Reason for exclusion
Albarqouni et al. 2018<br>*Evidence-based practice educational intervention studies: a systematic review of what is taught and how it is measured*	Publication type:<br>Not comprehensive literature search<br>No quality appraisal of included studies
Fiset et al. 2017<br>*Evidence-based practice i clinical nursing education: A scoping review*	Publication type:<br>No quality appraisal of included studies
Melender et al. 2016<br>*A systematic review on educational interventions for learning and implementing evidence-based practice in nursing: The state of evidence*	Population:<br>Also includes studies among postgraduate students and does not distinguish between level of education in the results section
Hitch et al. 2017<br>*Instructional practices for evidence-based practice with pre-registration allied health students: a review of resent research and developments*	Population:<br>Also includes studies among undergraduate/ baccalaureate students from other healthcare disciplines (e.g., paramedicine, social work) and studies among professionals (e.g., clinical instructors, faculty, librarians), or the population is not stated besides discipline and does not distinguish between level of education in the results section
Erichsen et al. 2016<br>*Kunnskapsbasert praksis i sykepleierutdanningen*	Publication type:<br>No quality appraisal of included studies
Larsen et al. 2019<br>*Methods for teaching evidence-based practice: a scoping review*	Publication type:<br>No quality appraisal of included studies
Aglen 2016<br>*Pedagogical strategies to teach bachelor students evidence-based practice: a systematic review*	Publication type:<br>No quality appraisal of included studies
Kyriakoulis et al. 2016	Publication type:<br>No quality appraisal of included studies

Educational strategies for teaching evidence-based practice to undergraduate health students: systematic review	
