# Supplementary material for: Interventions, methods and outcome measures used in teaching evidence-based practice to healthcare students: an overview of systematic reviews
Source: BMC Med Educ. 2024 Mar 19;24:306. doi: 10.1186/s12909-024-05259-8 (PMC10953117; doi:10.1186/s12909-024-05259-8)
Supplement: Supplementary file 3 — Supplementary Material 3. [file 12909_2024_5259_MOESM3_ESM.pdf]

Additional file 3. Primary studies in the systematic reviews that are excluded from this overview including reason for exclusion

Study ID: Review: Primary studies	Reason for exclusion
**Wakibi et al. :**	
Mattila et al. 2013	Population are RN to BSN students. Being a qualified nurse before aiming for a Bachelor Degree constitute a significantly different prerequisite basis than traditional Professional Bachelor Degree students in nursing
Oh et al. 2010	RN to BSN student (see above)
**Horntvedt et al. 2018:**	
Malik et al. 2017	Population are Nurse Academics
**Patelarou et al. 2020:**	
Liou et al. 2013	RN to BSN student (see above)
Hsieh et al. 2016	RN to BSN student (see above)
Singleton 2017	Population is Doctor of Nursing students
Hagler et al. 2012	Population is Nurse Preceptors
Justham and Timmons 2005	Population is post-registration Nursing students. Whether this is one week or three years after becoming a registered nurse is not clarified
Foronda et al. 2017	Population is master’s entry level Nursing students. Whether this is a summer holiday or ten years after registration as a nurse is not clarified
Rojjanasrirat and Rice 2017	Population is Nursing master students
**Cui et al. 2018**	
Fan M. 2013	Population is higher-vocational students. Contacting Cui et al. did not bring any further clarification to this type of students
Huang L.P. 2011	Population is a mixture of undergraduate and higher-vocational students
Jiang W.L. 2012	Population is (direct quote) “unknown”
Liu L.Z. 2016	Population is a mixture of postgraduate, undergraduate and higher-vocational students
Ma G.P. 2012	Population is higher-vocational students (see above)
**Ramis et al. 2019**	
Liabsuetrakul et al. 2009	Population is medical students
Liabsuetrakul et al. 2013	Population is medical students
