# Supplementary material for: Interventions, methods and outcome measures used in teaching evidence-based practice to healthcare students: an overview of systematic reviews
Source: BMC Med Educ. 2024 Mar 19;24:306. doi: 10.1186/s12909-024-05259-8 (PMC10953117; doi:10.1186/s12909-024-05259-8)
Supplement: Supplementary file 4 — Supplementary Material 4. [file 12909_2024_5259_MOESM4_ESM.pdf]

Additional file 4. Primary study overlaps within the included systematic reviews in the overview. Full documentation of primary studies (included in the six systematic reviews) regarding research designs, type of interventions, type of participants (student type), sample size and country and which systematic reviews each primary study is included in.

**Primary studies included in systematic reviews**						**Systematic reviews included in the overview**					
Author et al., year	Type of study design	Inter-vention	Partici-pants	Sample size	Country	Wakabi et.al. 2021	Ghaffari et al. 2018	Hornt-vedt et al. 2018	Patela-rou et al. 2020	Ramis et al. 2019	Cui et al. 2018
Brooke et al. 2015	Qual	MI	N	70	UK/ Slovenia	X					
Cosme et al. 2018	BA	MI	N	57	USA	X					
Dawley et al. 2011	Qual	MI	N	198	USA	X					
Finotto et al. 2013	CS	MI	N	300	Italy	X					
Foss et al. 2014	MM	MI	N	38	Norway	X					
Jalali-Nia et al. 2011	QE	MI	N	41	Iran	X					
Keib et al. 2017	BA	MI	N	109	USA	X			X		
Kim et al. 2009	QE	MI	N	208	USA	X			X	x	

Leach et al. 2016	BA	MI	N	33	Australia	X			X		
Reid et al. 2017	BA	MI	N/M	56	UK	X			X		
Ruzafa-Martinez et al. 2016	QE	MI	N	148	Spain	X			X		
Smith-Strom and Nortvedt 2008	MM	MI	N	48	Norway	X					
Zhang et al. 2012	BA	MI	N	74	China	X			X		
Madarshahian et al. 2011	QE	NS	N	40	Iran		X				
Khajeali et al. 2013	QE	NS	N	48	Iran		X				
Poudineh Moghadam et al. 2012	QE	NS	N	42	Iran		X				
Poudineh Moghadam et. Al. 2015	QE	NS	N	43	Iran		X				
Madarshahian et al. 2014	QE	NS	N	78	Iran		X				
Madarsharhian et al. 2012	QE	NS	N	40	Iran		X				

Hassanabadi et al. 2014	QE	NS	N	76	Iran		X				
Hassanabadi et al. 2013	QE	NS	N	36	Iran		X				
Andre' et al. 2016	Qual	MI	N	5	Norway			X			
Cader et al. 2006	Qual	SI	N	16	UK			X			
Friberg and Lyckhage 2013	Qual	MI	N	80	Sweden			X			
Irvine et al. 2008	MM	MI	N	49	UK			X			
Mattila and Eriksson 2007	Qual	SI	N	50	Finland			X			
Nayda and Rankin 2008	Qual	NS	N	394	Australia			X			
Scurlock-Evans et al. 2017	BA	MI	N	56	UK				X		
Kim et al. 2019	QE	MI	N	44	Korea				X		
Dias and Walsh, 2018	QE	SI	N	32	USA				X		
Whittaker, 2015	QE	MI	N	184	USA				X		

Mena-Tudela et la. 2018	QE	MI	N	83	Spain				X		
Oh et al. 2019	QE	MI	N	45	Korea				X		
Ashktorab et al. 2014	QE		N	80	Iran					x	
Long et al. 2016	QE/RCT	MI	NH	58	USA/Le-banon				X	x	
He et al. 2016	RCT	MI	N	90	China						x
Jiang et al. 2006	RCT	MI	N	120	China						x
Xie, X.Y., 2012	NS	MI	N	115	China						x
Zhong et al. 2016	RCT	MI	N	110	China						x

Type of study design

CS – Cross sectional survey

QE - Quasi experimental study

Qual - Qualitative study

MM - Multi Method (qualitative and quantitative methods used)

BA - Before and after study

RCT- Randomized Controlled Trial

NS – Not specified

Intervention

SI – Single Intervention (covering one educational activity)

MI – Multifaceted intervention (covering a combination of educational activities)

NS - Not Specified

Participants

N - Nurse students

NH – Nutrition and Health students

M – Midwifery students
